# Prevalence and Prognostic Factors of Stress Hyperglycemia in a Pediatric Population with Acute Illness in Greece—A Prospective Observational Study

**DOI:** 10.3390/jcm11051301

**Published:** 2022-02-27

**Authors:** Emmanouil Korakas, Theodoros Argyropoulos, Georgia-Angeliki Koliou, Aristofanis Gikas, Aikaterini Kountouri, Stavroula Kostaridou Nikolopoulou, Panagiotis Plotas, Konstantinos Kontoangelos, Ignatios Ikonomidis, Nikolaos P. E. Kadoglou, Athanasios Raptis, Vaia Lambadiari

**Affiliations:** 1 Second Department of Internal Medicine and Research Institute, Attikon University Hospital, Medical School, National and Kapodistrian University of Athens, 12462 Athens, Greece; mankor-th@hotmail.com (E.K.); gkoliou@buffalo.edu (G.-A.K.); katerinak90@hotmail.com (A.K.); atraptis@med.uoa.gr (A.R.); 2 Department of Paediatrics, Penteli Children’s Hospital, 15236 Athens, Greece; argitheo@gmail.com (T.A.); vkostaridou@gmail.com (S.K.N.); 3 Health Center of Kalivia, 19010 Athens, Greece; aristofanisg@yahoo.gr; 4 Department of Cardiology, University of Patras Medical School, 26504 Patras, Greece; pplotas@upatras.gr; 5 First Department of Psychiatry, Eginition Hospital, Medical School, National and Kapodistrian University of Athens, 11528 Athens, Greece; kontoangel@med.uoa.gr; 6 Second Cardiology Department, Attikon University Hospital, Medical School, National and Kapodistrian University of Athens, 12462 Athens, Greece; ignoik@gmail.com; 7 Medical School, University of Cyprus, Nicosia 1678, Cyprus; nikoskad@yahoo.com

**Keywords:** stress hyperglycemia, asthma, corticosteroids, β2-agonists, pediatric

## Abstract

Background: stress hyperglycemia (SH) is a relatively frequent finding in pediatric patients. The purpose of this prospective observational study was to identify the prevalence of pediatric SH and its associated risk factors in Greece. Methods: A total of 1005 patients without diabetes who were admitted consecutively for acute illness in a Pediatric Emergency Department were included in the study. Medical history, anthropometric measurements, blood glucose levels, and the medication administered were recorded. A questionnaire was distributed to parents regarding medical and perinatal history and sociodemographic characteristics. Results: There were 72 cases of SH on admission (7.2%) and 39 (3.9%) during hospitalization. Mean age was 6.4 years; 50.3% were male. SH on admission was associated with oral corticosteroid therapy (21.1% vs. 4.7%, *p* < 0.001), inhaled corticosteroids (12.7% vs. 3%, *p* < 0.001), and inhaled β2-agonists (30.6% vs. 10.7%, *p* < 0.001). In-hospital hyperglycemia was associated with oral corticosteroids (adjusted OR = 3.32), inhaled corticosteroids (OR = 10.03) and inhaled β2-agonists (OR = 5.01). Children with asthma were 5.58 and 7.86 times more likely to present admission and in-hospital hyperglycemia, respectively. Conclusions: This is the first report of SH prevalence in pediatric patients in Greece. Asthma, corticosteroids, and β2-agonists significantly increase the risk of SH. No parental factors seem to predispose to SH.

## 1. Introduction

According to the latest American Diabetes Association and American Association of Clinical Endocrinologists consensus, stress hyperglycemia (SH) is defined as any transient inpatient plasma glucose levels >140 mg/dL under conditions of acute physical or psychological stress, without evidence of previous diabetes [1]. In the majority of cases, these abnormal values return to normal as soon as the stressful trigger has passed. Stress hyperglycemia has long been considered a normal homeostatic response to acute stress, and it is mainly mediated through the elevated production of counter-regulatory hormones such as cortisol, glucagon, growth hormone, catecholamines, and various cytokines, which stimulate glycogenolysis and gluconeogenesis in an insulin-independent manner [2,3]. Hepatic and peripheral insulin resistance, increased lipolysis, relative insulin deficiency, and decreased glucose cellular uptake due to pro-inflammatory molecules such as interleukin 1 (IL-1), interleukin 6 (IL-6), and tumor necrosis factor α (TNF-α), may also contribute.

The incidence of SH has been reported to be as high as 5% in children presenting to pediatric emergency departments (EDs), with blood glucose concentrations ranging between 150 and 299 mg/dL in most cases [4]. In more critical illness and, especially, in the intensive care unit (ICU) setting, incidence can reach as high as 60%, and it has been estimated that glucose levels above 200 mg/dL occur in 20–35% of critically ill children [5,6]. Various conditions have been associated with increased risk of SH, with most cohorts reporting a higher risk for respiratory infections, febrile seizures, gastrointestinal disorders, trauma, and cardiac surgery. However, there is a paucity of data regarding possible associations of SH with factors other than the underlying disease, such as sociodemographic factors or perinatal history, especially regarding the Greek population, where no data are available. The aim of this study was to determine the incidence of SH in a Greek cohort of patients admitted to the ED of a tertiary pediatric referral center in Athens, and to investigate its causes and possible risk factors regarding both patients themselves and parental characteristics.

## 2. Materials and Methods

### 2.1. Study Design and Population

We conducted a prospective observational study of pediatric and adolescent patients who were admitted to the Emergency Department of Penteli’s Children Hospital, which is a big referral center covering a broad area with a diverse population, and were hospitalized in the respective pediatric clinics due to acute illness, from 1 May 2017 to 30 June 2020. Children with age of <1 year old, children with diabetes mellitus, and children whose parents were not fluent in Greek or presented signs of cognitive disorders were excluded. Anthropometric measurements including weight, height, waist circumference, and body mass index (BMI), along with vital signs (arterial pressure, heart rate, body temperature, and oxygen saturation) were recorded in all patients enrolled. A complete medical history was obtained. Blood glucose values, both on admission and during hospitalization, were recorded together with any other laboratory data obtained and medications administered both in the ED and during hospitalization. A structured questionnaire regarding medical history, perinatal history, and sociodemographic characteristics was distributed to parents (Table 1). An informed consent form was obtained by all patients’ parents included in the study.

### 2.2. Data Analysis

Continuous variables were expressed as the mean ± standard deviation (SD) and categorical variables were reported as percentages with the corresponding percentages. Differences in continuous variables were evaluated using the parametric Student *t*-test given the (approximately) normal distribution of the examined variables. The chi-square or Fisher’s exact test (if more appropriate) was used for group comparisons of categorical data.

Univariate logistic regression models were initially applied to examine the association of certain variables of interest with the presence of hyperglycemia at admission and during hospital stay. Subsequently, multivariate analysis was performed adjusting for age and for all variables that were associated with hyperglycemia in the univariate analysis. Analyses were performed using SPSS 21.0 and statistical significance was set at two-sided *p* < 0.050.

## 3. Results

### 3.1. Patient Characteristics

Overall, 1005 consecutive patients, aged 1–16 years old (median 5), were included. Mean age was 6.4 years (±4.2); 50.3% were males. In 591 cases (58.8%) caesarean section was the method of delivery. In total, 871 children (86.7%) were full-term, and the mean birth weight for 924 children with available information was 3092.8 ± 487.2 g (range 770–4400 g). Breastfeeding (not exclusive) for at least 3 months had taken place in 699 cases (69.6%). Regarding body weight, approximately half of the children (*n* = 498, 49.6%) were within normal BMI limits, while 321 children (31.9%) were considered underweight. Mean duration of hospital stay was 3.7 (± 2.2) days (range 1–20 days) and most children were hospitalized for 1–6 days (*n* = 923, 91.8%). No deaths were reported during the duration of hospitalization. The mean maternal age at birth was 31.3 ± 5.8 years (Table 2).

### 3.2. Stress Hyperglycemia and Associated Risk Factors

There were 72 cases of SH on admission (7.2%) and 39 (3.9%) during hospitalization. The mean value of BG on admission was 94.2 ± 22.5 mg/dL and the mean value during hospitalization was 92.3 ± 17.6 mg/dL. Children who had stayed in hospital for more than 6 days had higher admission as well as in-hospital BG levels compared with those who had stayed 1–6 days (99.1 ± 23.6 vs. 93.7 ± 22.4, *p* = 0.039 and 97.07 ± 13.0 vs. 91.9 ± 17.9, *p* = 0.010, respectively).

Children with SH at admission compared to those without had more frequently received oral corticosteroid therapy (21.1% vs. 4.7%, *p* < 0.001), inhaled corticosteroids (12.7% vs. 3%, *p* < 0.001), and inhaled β2-agonists (30.6% vs. 10.7%, *p* < 0.001). More specifically, children who had received oral corticosteroids were 5.41 times more likely to present SH at admission, while the odds of hyperglycemia at admission were significantly higher for children who had received inhaled corticosteroids (odds ratio (OR) = 4.69, 95% confidence interval (CI) 2.12–10.37, *p* < 0.001) as well as for those treated with inhaled β2-agonists (OR = 3.66, 95% CI 2.13–6.30, *p* < 0.001). Similarly, in-hospital hyperglycemia was associated with the administration of oral corticosteroids (OR = 4.63, *p* < 0.001), inhaled corticosteroids (OR = 10.03, *p* < 0.001) and inhaled β2-agonists (OR = 5.01, *p* < 0.001) (Table 3 and Table 4).

**Figure 1:**
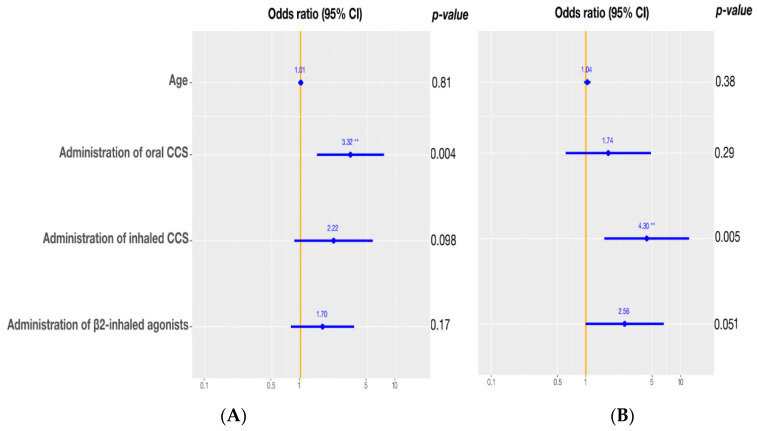
Risk factors for admission and in-hospital stress hyperglycemia after multivariate analysis. (**A**) Admission hyperglycemia. (**B**) In-hospital hyperglycemia. ** *p* < 0.001.

Additionally, a significant association was revealed between the presence of hyperglycemia at admission and during hospitalization and the admission due to asthma (both *p*-values < 0.001), with children admitted to hospital due to asthma being 5.58 and 7.86 times more likely to present hyperglycemia at admission and in-hospital, respectively (Table 3 and Table 4). Of note, none of the children admitted to hospital due to pneumonia presented with hyperglycemia during hospitalization. Upon adjustment for children’s age, administration of oral corticosteroids remained a significant predictor for hyperglycemia at admission (OR = 3.32, *p* = 0.004), while only a trend towards higher odds of admission hyperglycemia was observed for children treated with inhaled corticosteroids (OR = 2.22, *p* = 0.098). Administration of β2-inhaled agonists did not retain its significance in the multivariate model. However, the estimated odds ratio for the presence of hyperglycemia at admission remained in the same direction as the one obtained in the univariate analysis (OR = 1.70, *p* = 0.17) (Figure 1A).

In terms of in-hospital hyperglycemia, administration of inhaled corticosteroids remained a significant predictor in the multivariate analysis (OR = 4.30, *p* = 0.005), whereas treatment with β2-inhaled agonists was marginally significantly associated with hyperglycemia during hospitalization (OR = 2.56, *p* = 0.051) and treatment with oral corticosteroids lost its statistical significance (OR = 1.74, *p* = 0.29) after adjustment for age and the aforementioned parameters (Figure 1B).

Age did not show predictive significance in any of the examined models.

## 4. Discussion

This is the first study which reports the prevalence of pediatric stress hyperglycemia in Greece. We have shown that the incidence of stress hyperglycemia in children visiting a pediatric Emergency Department was 7.2% on admission and 3.9% during hospitalization. This incidence is comparable to previous data from various territories [7], such as these by Valerio et al. (4.9%) or Gupta et al. (4.7%) [8,9]. A possible reason for the difference between admission and in-hospital rates could be that the referral center in our study does not treat major trauma and it does not host patients subjected to cardiac surgery, which are two conditions that have been significantly associated with SH in previous studies [8,10,11].

Consistent with prior reports, SH was documented in a variety of acute conditions frequently encountered in the ED, such as respiratory infections, CNS disorders, gastrointestinal disorders and asthma exacerbations [4,12,13,14]. The statistically significant results, however, were shown for asthma, where it was demonstrated that the disease itself, together with the use of corticosteroids, either oral or inhaled, or the administration of inhaled β2-agonists was significantly associated with SH both on admission and during hospitalization. In general, respiratory disorders have been determined as a major risk factor for SH in various reports. In a study by Faustino et al. [5], pulmonary diseases comprised 16.8% of the total medical conditions associated with SH in a pediatric population, while an even greater rate of 37.8% was reported by Levmore-Tamir et al. [12] in a large retrospective cohort of 1245 cases with SH. Most remarkable were the results by Weiss et al. [10], where cases of extreme SH (>300 mg/dL) were studied; respiratory illness was again the most common diagnosis, accounting for 49% of the patient visits. The association of corticosteroids with glucose dysregulation has been well-established [15]. The main mechanism is the reduction in insulin sensitivity, which has been observed both during intravenous infusion and oral administration, in a manner which has also proven to be dose-dependent [16]. Corticosteroids increase hepatic gluconeogenesis by activating genes for glucose-6-phosphatase and by upregulating the counter-regulatory hormones such as glucagon and adrenaline; on the other hand, they also suppress the compensatory action of increased insulin secretion at higher doses. The relation of β2-agonists to hyperglycemia has long been known as well; in a report by Dawson et al., where 12 children of a mean age of 60.8 months were studied, glucose levels were significantly elevated within 2 h of nebulized salbutamol administration [13]. More recently, Mobaireek et al. studied 166 children admitted with acute asthma; hyperglycemia was observed in 38.6% of the patients, and an inverse association between K+ and HCO3-levels and blood glucose was noted, a finding which further supported the notion that SH was attributed to the use of β2-agonists [17]. The stimulation of the β2 receptor results in increased hepatic and muscle glycogenolysis and gluconeogenesis, however the hyperglycemic effect of β2-agonists has not yet been fully elucidated. On the contrary, corticosteroid-induced hyperglycemia is the cumulative result of multiple mechanisms, including increased hepatic gluconeogenesis, upregulation of counter-regulatory hormones such as glucagon and epinephrine, and insulin resistance, with these effects taking place in a dose-dependent manner [18].

Febrile gastroenteritis was documented in 10.3% of the total cases of SH, a prevalence which is comparable to that described in previous reports; contrary to these data, however, no statistically significant association was established. In the study by Levmore-Tamir et al. [12], the respective prevalence was 14.1%. In an 8-month prospective study in a diarrhea treatment center in Bangladesh, prevalence of hyperglycemia among patients aged 2–10 years old was 9.4% [14]. Severe dehydration is possibly the trigger factor for hyperglycemia, through mechanisms which include decreased renal perfusion and subsequent decreased urinary glucose excretion, along with activation of counter-regulatory hormones such as adrenaline and cortisol to preserve sufficient circulatory blood volume and arterial pressure [19,20]. A possible explanation for these results is probably the very low number of events in this group, as shown in Table 4.

Similarly, no significant association was demonstrated between SH and CNS disorders, especially seizures, in our cohort, again contrary to previously published data [12]. This finding is relatively unexpected, as febrile seizures have been considered one of the most prominent risk factors for SH, with its incidence ranging from 10% to 16% [8,10,20]. In the most recent report by Iflah et al. [21], in children hospitalized for acute gastroenteritis, high blood glucose level was positively associated with convulsions (OR 5.71, *p* = 0.023), although it was noted that a firm causal relationship could not be established. Possible explanations for this discrepancy include the relatively small number of patients presenting with seizures in our cohort, along with the fact that the major determinants of SH prevalence seem to be the severity and duration of seizures, rather than the presence of seizures per se. In our cohort, no severe seizure events were documented. In the same notion, despite the fact that a high percentage of children presented with fever in our sample (62%), no association between body temperature and SH was shown, a finding which can be attributed to the fact that fever was not accompanied by seizures or pain. In the study by Valerio et al. [8], the stress hyperglycemia prevalence of 4.9% in children with febrile seizures was higher than that in children with fever alone (4.4%) and, similarly, in the study by Lee et al. [22], variables associated with fever did not exert any impact on the incidence of SH.

As expected, apart from the aforementioned asthma treatments, we found no association between medication during hospitalization and SH. Treatment options which have an established relationship to SH, such as vasopressors, parenteral nutrition, or mechanical ventilation, refer to ICU settings [4,23,24,25] and were therefore not used in our cohort as the hospital in our study lacks a pediatric ICU. Furthermore, no association was found between SH and age, gender, BMI, childbirth method, or other sociodemographic factors, in accordance with all the aforementioned studies. A possible association between age and SH has been noted only in the reports by Karamifar et al. [26] and Bordbar et al. [27], where age < 6 years old was associated with increased SH prevalence for reasons not clarified, while in the report by Ognibene et al. [28], hyperglycemia was more frequently documented in patients > 13 years who, however, also had a more serious underlying condition.

No statistically significant association was found regarding parental sociodemographic characteristics and medical history, a finding which is consistent with the vast majority of studies in the field. An exception to this pattern was a report from a special baby care unit in Nigeria, where parental low socioeconomic class, vaginal delivery, and grand multiparity were associated with higher SH prevalence [29]. However, the study was conducted on neonatals (age < 28 days) and, therefore, the results cannot be extrapolated to children of our age range. In a small study with 60 critically ill children and 21 healthy controls, stress hyperglycemia was negatively correlated with the age of patients (r = −0.305), but no statistically significant correlation between glucose levels and length of hospital stay was shown [30]. Similarly, only one report has pointed out a relationship between SH and family history of diabetes mellitus (DM) [27].

The primary strength of our study is that it included all consecutive patients who were admitted to the ED during the study period, which led to a large study sample with limited selection bias. A significant limitation is the absence of patients with major trauma or cardiac surgery, which are conditions frequently associated with SH, while, on the other hand, the majority of patients in our cohort suffered from respiratory infections. No data were obtained regarding glycosylated hemoglobin or cases that were possibly subsequently diagnosed with overt diabetes. Data regarding the time that the last meal of the patients had taken place before glucose measurement were not obtained, so whether patients were in a postprandial state on admission is unknown. However, glucose values > 140 mg/dL are not expected even immediately after meal in healthy children, so it is logical to suggest that all cases of hyperglycemia can be attributed to SH.

## 5. Conclusions

Our study aimed to examine the prevalence of stress hyperglycemia and its possible causes in acutely ill children. This is the first report in the Greek population, which is highly representative of the Mediterranean region, with a specific prospective design. Asthma is the disease which is most commonly associated with stress hyperglycemia, while, in terms of medications, corticosteroids, regardless of administration route, and β2-agonists are the medicinal agents which significantly increase the risk for SH. No medical or sociodemographic factors of the parents seem to increase the risk for SH. This study confirms previous data about the possible causes of stress hyperglycemia, but as some results were rather surprising, it urges the need for more large-scale studies in different healthcare settings to further clarify possible risk factors for SH in the pediatric population, both at the individual and parental level. In any case, awareness should be raised, especially, according to our findings, for children presenting with respiratory illness, so that early detection is achieved.

## Figures and Tables

**Table 1:** Questionnaire regarding parental factors. BMI: body mass index; BG: blood glucose; DM: diabetes mellitus; and IVF: in vitro fertilization.

Variable (Units)	Answers
Education of mother	Low/moderate/higher
Maternal occupation	Employed/unemployed
Age of mother (years)	Free answer
Age of mother at birth (years)	Free answer
BMI of mother (kg/m^2^)	<25/25–29.9/≥30
BG of mother (mg/dL)	<95/96–110/>110
Residency	Urban/suburban/rural
Family history of DM	Yes/No
Thyroid disorder	Yes/No
History of smoking	Yes/No
Mediterranean Diet	Yes/No
Childbirth method	Natural/Caesarean section
Weight gain during pregnancy (kg)	<12/≥12
Maturity at birth	Full-term/premature
Weight at birth (kg)	Free answer
Breastfeeding	Yes/No
Method of fertilization	Natural/IVF
Gestational diabetes	Yes/No
Complications during labor	Yes/No
History of miscarriages	Yes/No

**Table 2:** Characteristics of the mothers and the children. BG: Blood Glucose; BMI: Body Mass Index, and CNS: Central Nervous System. Values are presented as *n* (column %) or mean (standard deviation).

Characteristics	*n* (%)
**Mothers**	
Age of mother at birth (*n* = 1002)	31.3 ± 5.8 (range 14–53)
**Children**	
Childbirth method (*n* = 1005)	
Natural	414 (41.2)
Caesarean Section	591 (58.8)
Maturity at birth (*n* = 1005)	
Full term	871 (86.7)
Premature	134 (13.3)
Weight at birth (grams) (*n* = 924)	3092.8 ± 487.2 (range 770–4415)
Breastfeeding (*n* = 1005)	
No	306 (30.4)
Yes	699 (69.6)
BMI (kg/m^2^) (*n* = 1005)	
Underweight	321 (31.9)
Normal	498 (49.6)
Overweight	125 (12.4)
Obese	61 (6.1)
Causes of admission (*n* = 1005)	
Upper respiratory infections	114 (11.3)
Pneumonia	118 (11.7)
Asthma	57 (5.7)
Gastroenteritis	107 (10.6)
Viral infections	129 (12.8)
CNS disorders	41 (4.1)
Skin and subcutaneous infections	44 (4.4)
Bacteremia	53 (5.3)
Fever of unknown origin	61 (6.1)
Other disorders	281 (28.0)
Mean length of hospital stay (*n* = 1005) 3.7 ± 2.2 (range 1–20)	

**Table 3:** Association of admission and in-hospital hyperglycemia with selected parameters of interest. std: standard deviation; CCS: Corticosteroids; ^a^: *t*-test; and ^b^: chi-square/Fisher’s exact test. Significant *p*-values are shown in bold. ^ continuous variable.

	Admission Hyperglycemia			In-Hospital Hyperglycemia		
Parameter	No	Yes	*p*-Value	No	Yes	*p*-Value
Age ^, mean ± std	6.4 ± 4.2	6.6 ± 4.2	0.77 ^a^	6.4 ± 4.2	7.1 ± 3.9	0.28 ^a^
Maternal age at birth ^, mean ± std	31.4 ± 5.8	30.5 ± 5.3	0.20 ^a^	31.4 ± 5.8	31.0 ± 5.2	0.70 ^a^
	***n* (%)**	***n* (%)**		***n* (%)**	***n* (%)**	
Gender			0.58 ^b^			0.13 ^b^
Male	472 (50.6)	34 (47.2)		491 (50.8)	15 (38.5)	
Female	461 (49.4)	38 (52.8)		475 (49.2)	24 (61.5)	
Maturity at birth			0.56 ^b^			
Premature	807 (86.5)	64 (88.9)		833 (86.2)	38 (97.4)	--
Full-term	126 (13.5)	8 (11.1)		133 (13.8)	1 (2.6)	
Received oral CCS			**<0.001 ** ^**b**^			**<0.001 ** ^**b**^
No	889 (95.3)	56 (78.9)		914 (94.7)	31 (79.5)	
Yes	44 (4.7)	15 (21.1)		51 (5.3)	8 (20.5)	
Received inhaled CCS			**<0.001 ** ^**b**^			**<0.001 ** ^**b**^
No	904 (97.0)	62 (87.3)		936 (97.1)	30 (76.9)	
Yes	28 (3.0)	9 (12.7)		28 (2.9)	9 (23.1)	
Received β2-inhaled agonists			**<0.001 ** ^**b**^			**<0.001 ** ^**b**^
No	832 (89.3)	50 (69.4)		858 (88.9)	24 (61.5)	
Yes	100 (10.7)	22 (30.6)		107 (11.1)	15 (38.5)	
Admission due to upper respiratory infection			0.22			0.46
No	824 (88.3)	67 (93.1)		855 (88.5)	36 (92.3)	
Yes	109 (11.7)	5 (6.9)		111 (11.5)	3 (7.7)	
Admission due to pneumonia			0.56			0.020
No	825 (88.4)	62 (86.1)		848 (87.8)	39 (100.0)	
Yes	108 (11.6)	10 (13.9)		118 (12.2)	0 (0.0)	
Admission due to asthma			**<0.001**			**<0.001**
No	891 (95.5)	57 (79.2)		920 (95.2)	28 (71.8)	
Yes	42 (4.5)	15 (20.8)		46 (4.8)	11 (28.2)	
Admission due to Gastroenteritis			0.19			0.94
No	837 (89.7)	61 (84.7)		863 (89.3)	35 (89.7)	
Yes	96 (10.3)	11 (15.3)		103 (10.7)	4 (10.3)	
Admission due to viral infections			0.65			0.63
No	812 (87.0)	64 (88.9)		843 (87.3)	33 (84.6)	
Yes	121 (13.0)	8 (11.1)		123 (12.7)	6 (15.4)	
Admission due to CNS disorders			0.058			0.74
No	898 (96.2)	66 (91.7)		927 (96.0)	37 (94.9)	
Yes	35 (3.8)	6 (8.3)		39 (4.0)	2 (5.1)	

**Table 4:** Odds ratios estimated by univariate multivariate logistic regression models. CI: Confidence Interval; CCS: Corticosteroids. ^ continuous variable. * Odds ratios were not calculated due to the extremely small number of the events of interest in the group of full-term children. ** Odds ratios were not calculated due to absence of the event of interest in the group of children with pneumonia. Significant *p*-values are shown in bold.

	Admission Hyperglycemia			In-Hospital Hyperglycemia		
Parameter	Event/Total	Odds Ratio (95% CI)	*p*-Value	Event/Total	Odds Ratio (95% CI)	*p*-Value
Age ^	--	1.01 (0.95–1.07)	0.77	--	1.04 (0.97–1.12)	0.28
Maternal age at birth ^	--	0.97 (0.93–1.01)	0.20	--	0.99 (0.94–1.05)	0.70
Gender						
Male	34/506	[Reference]	--	15/506	[Reference]	--
Female	38/499	1.14 (0.71–1.85)	0.58	24/499	1.65 (0.86–3.19)	0.13
Maturity at birth						
Premature	64/871	[Reference]	--	38/871	[Reference]	--
Full-term	8/134	1.25 (0.59–2.67)	0.57	1/134	-- *	
Oral CCS						
No	56/945	[Reference]	--	31/945	[Reference]	--
Yes	15/59	5.41 (2.84–10.32)	**<0.001**	8/59	4.63 (2.02–10.57)	**<0.001**
Inhaled CCS						
No	62/966	[Reference]	--	30/966	[Reference]	--
Yes	9/37	4.69 (2.12–10.37)	**<0.001**	9/37	10.03 (4.35–23.10)	**<0.001**
β2-inhaled agonists						
No	50/882	[Reference]	--	24/882	[Reference]	--
Yes	22/122	3.66 (2.13–6.30)	**<0.001**	15/122	5.01 (2.55–9.85)	**<0.001**
Upper respiratory infection						
No	67/891	[Reference]	--	36/891	[Reference]	--
Yes	5/114	0.56 (0.22–1.43)	0.23	3/114	0.64 (0.19–2.12)	0.47
Pneumonia						
No	62/887	[Reference]	--	39/887	[Reference]	--
Yes	10/118	1.23 (0.61–2.48)	0.56	0/118	--	-- **
Asthma						
No	57/948	[Reference]	--	28/948	[Reference]	--
Yes	15/57	5.58 (2.92–10.67)	**<0.001**	11/57	7.86 (3.68–16.76)	**<0.001**
Gastroenteritis						
No	61/898	[Reference]	--	35/898	[Reference]	--
Yes	11/107	1.57 (0.80–3.09)	0.19	4/107	0.96 (0.33–2.75)	0.94
Viral infections						
No	64/876	[Reference]	--	33/876	[Reference]	--
Yes	8/129	0.84 (0.39–1.79)	0.65	6/129	1.25 (0.51–3.04)	0.63
CNS disorders						
No	66/964	[Reference]	--	37/964	[Reference]	--
Yes	6/41	2.33 (0.95–5.75)	0.065	2/41	1.29 (0.30–5.53)	0.74

## Data Availability

Study data is available upon reasonable request.

## Abbreviations

BG	blood glucose
BMI	body mass index
CCS	corticosteroids
CIs	confidence interval
CNS	central nervous system
DM	diabetes mellitus
ED	emergency department
FBG	fasting blood glucose
ICU	intensive care unit
IL-1	interleukin 1
IL-6	interleukin 6
OR	odds ratio
SH	stress hyperglycemia
TNF-α	tumor necrosis factor α
URI	upper respiratory infections
